# Changes in Treatment Patterns and Costs for Lung Cancer Have Not Resulted in Relevant Improvements in Survival: A Population-Based Observational Study in Catalonia

**DOI:** 10.3390/cancers14235791

**Published:** 2022-11-24

**Authors:** Laura Guarga, Noelia Paco, Emili Vela, Montse Clèries, Julieta Corral, Joaquim Delgadillo, Caridad Pontes, Josep Maria Borràs

**Affiliations:** 1 Servei Català de la Salut (CatSalut), 08007 Barcelona, Spain; 2 Departament de Farmacologia, de Terapèutica i de Toxicologia, Universitat Autònoma de Barcelona, 08193 Barcelona, Spain; 3 Digitalization for the Sustainability of the Healthcare System (DS3), Bellvitge Biomedical Research Institute (IDIBELL), 08006 Barcelona, Spain; 4 Pla Director d’Oncologia, Departament de Salut, Hospitalet del Llobregat, 08908 Barcelona, Spain; 5 Bellvitge Biomedical Research Institute (IDIBELL), 08006 Barcelona, Spain; 6 Banc de Sang i Teixits (BST), 08005 Barcelona, Spain; 7 Departament de Ciències Clíniques, Universitat de Barcelona, Campus de Bellvitge, 08907 Barcelona, Spain

**Keywords:** lung cancer, real-world data, immunotherapy, targeted therapy, overall survival, cost of treatment

## Abstract

**Simple Summary:**

Real-world data collect clinical and economic information from daily clinical practice and can support decisions in the context of health evaluation and management. The aim of our retrospective cohort study was to describe the different approaches used for treating lung cancer in Catalonia in 2014 and 2018 and to assess the associated cost and impact on patient survival until December 2021. Treatment patterns for lung cancer changed in younger patients, and all costs of treatment increased significantly. These changes, mainly related to the use of several novel drugs, such as immunotherapy and targeted therapy, were not associated with an increase in the overall patient survival in the period of time under evaluation.

**Abstract:**

Objective: Few published studies have described multidisciplinary therapeutic strategies for lung cancer. This study aims to describe the different approaches used for treating lung cancer in Catalonia in 2014 and 2018 and to assess the associated cost and impact on patient survival. Methods: A retrospective observational cohort study using data of patients with lung cancer from health care registries in Catalonia was carried out. We analyzed change in treatment patterns, costs and survival according to the year of treatment initiation (2014 vs. 2018). The Kaplan–Meier method was used to estimate survival, with the follow-up until 2021. Results: From 2014 to 2018, the proportion of patients undergoing surgery increased and treatments for unresectable tumors decreased, mainly in younger patients. Immunotherapy increased by up to 9% by 2018. No differences in patient survival were observed within treatment patterns. The mean cost per patient in the first year of treatment increased from EUR 14,123 (standard deviation [SD] 4327) to EUR 14,550 (SD 3880) in surgical patients, from EUR 4655 (SD 3540) to EUR 5873 (SD 6455) in patients receiving curative radiotherapy and from EUR 4723 (SD 7003) to EUR 6458 (SD 10,116) in those treated for unresectable disease. Conclusions: From 2014 to 2018, surgical approaches increased in younger patients. The mean cost of treating patients increased, especially in pharmaceutical expenditure, mainly related to the use of several biomarker-targeted treatments. While no differences in overall patient survival were observed, it seems reasonable to expect improvements in this outcome in upcoming years as more patients receive innovative treatments.

## 1. Introduction

Lung cancer is the leading cause of cancer-related death in Europe, with an age-standardized mortality rate of 54.2 per a population of 100,000 [1]. Approximately 70% to 75% of patients are diagnosed at advanced stages [2,3], associated with a poor prognosis and five-year survival of around 15% in high-income countries [4]. In Spain, there were an estimated 29,188 new cases in 2020; in line with the incidence throughout Europe, it represents one of the five most frequently diagnosed cancers, together with breast, colorectal, prostate, and stomach cancers [1,5].

In 2018, the total cost of cancer care in Europe was estimated at EUR 199 billion, a figure expected to increase in the coming years, mainly as a result of new treatments [6,7]. In Spain, the total cost of cancer care in the same year was EUR 12,164 million, of which, 43% was spent on drugs [6]. The type of tumor requiring the most resources—approximately 15% of the total—was lung cancer, followed by breast, colorectal, and prostate cancer [8].

Upon diagnosis of lung cancer, the main therapeutic aim is to be able to perform a procedure with curative intent, such as complete surgery or curative radiotherapy, that includes either stereotactic body radiation (SBRT) or hypofractionated high-dose radiotherapy [9]. When the tumor is inoperable or unresectable, the therapeutic strategy is based on pharmacological treatments aiming to palliate symptoms and extend survival [10,11].

The prognosis of lung cancer has been reported to improve in certain patient subgroups, primarily due to an increased availability of new drugs [11,12]. From 2014 to 2021 in Spain, 25 pharmacological treatments, corresponding to 12 different molecules with different targets, were granted public reimbursement by the Spanish Health Service [13]. These new molecules, generally indicated for advanced tumor stages, represent 46% of all currently available active substances for treating lung cancer [13]. From 2014 to 2021, 14 out of 25 newly reimbursed treatments were tyrosine kinase inhibitors (TKIs) targeting tumors with *epidermal growth factor receptor* (EGFR) mutations, *anaplastic lymphoma kinase* (ALK) translocations and the *proto-oncogene tyrosine-protein kinase* (ROS-1), present in 1% to 11% of patients with advanced non-small cell lung carcinoma [14]. In 2016, the first second-line monotherapies with inhibitors of programmed death protein (PD1) or its ligand (PD-L1)—expressed in 24% to 60% of patients with lung adenocarcinoma and up to 83% of patients with small cell lung carcinoma—were also reimbursed [15]. From 2014 to 2021, immunotherapy accounted for 44% of the newly reimbursed oncological treatments (10 new indications).

In general, the clinical impact of new therapies needs to be evaluated in practice due to differences between research and actual clinical practice, including the selection of subjects and the drug sequences used in clinical trials [16,17,18]. Real-world data and/or observational studies in oncology are emerging as a useful tool for collecting data from daily clinical practice and supporting clinical decisions [16,17]. These can be complemented with an assessment of the budgetary impact and other additional relevant information, which can be potentially useful for decision making in the context of health evaluation and management [16,19,20,21]. This type of study expedites the availability of data, notably shortening the intervals compared to population-based studies using cancer registries.

Few published studies have described all of the treatment patterns used in clinical practice for lung cancer [2,22]; most mainly focus on one type of procedure [23], group of medications [16,24,25,26] or tumor subtype [27,28]. A comprehensive analysis of the progressive changes in the management of this pathology, including surgery, radiotherapy and pharmacological treatments, as well as their impact on both patients’ health and the public healthcare budget, could better inform decision making around the organization and financing of cancer care. This study aims to describe the different treatment patterns used for treating lung cancer in Catalonia in 2014 and 2018 and to assess the associated cost and impact on patient survival.

## 2. Methods

This observational study followed STROBE criteria [29] and used data from different Catalan healthcare registries for the 2014–2018 period to assess treatment patterns, treatment cost and survival outcomes in 2021.

### 2.1. Data Source

The Catalan Health Service (CatSalut) centrally manages all healthcare registries in Catalonia, collecting clinical practice data on 68 hospitals that provide public universal care to 7.7 million people [30]. For this study, we combined data from the following healthcare registries [30,31,32].

- The hospital discharge minimum basic data set, which collects information related to acute hospital care, including surgery;
- The hospital outpatient drugs registry, which contains clinical information on drugs prescribed from different therapeutic areas (not including chemotherapy treatments);
- The Catalan Health Surveillance System, which collects data on health services, including radiotherapy;
- The Datamart Billing Service, which collects all of the specific billing data for hospital outpatient drugs (including chemotherapy treatments);
- The Central Registry of Insured Persons, which collects basic demographic data of insured people covered by CatSalut.

Some relevant data were not available in the centralized registries, such as certain information related to the tumor (histology, staging, biomarkers), the patient (functional status, tobacco consumption) or the treatment (indication of chemotherapy or radiotherapy).

### 2.2. Study Population

The study included patients with a first diagnosis of lung cancer (International Classification of Diseases, 10th revision ICD-10-C34) [33] recorded in the hospital discharge minimum basic data set from 2014 to 2018 and with available information on vital status until December 2021. These periods were selected to enable identification of measurable changes in survival outcomes and costs, since the first immunotherapy regimens, immune checkpoint inhibitors (ICI) for second and first-line of treatment, were reimbursed in 2016 and 2017, respectively.

Data of treatment types were collected from the hospital discharge minimum basic data set, the Catalan health surveillance system, hospital outpatient drugs registry and Datamart Billing Service. To avoid double-counting patients treated in different hospitals, a unique anonymous identifier was created for each case, which enabled combining data for patients treated in more than one center.

A total of 15% to 17% of patients treated surgically received part of or all of the treatment in private practice, although some of these patients may return to the public system to receive systemic treatment. Only the information available from the public health care system was included in the analysis for these patients.

### 2.3. Outcomes

A descriptive analysis was performed on demographic characteristics by year of treatment initiation (2014 and 2018), including sex and age group (<60 years, 60–69 years, 70–79 years and ≥80 years). Treatment patterns initiated in 2014 and 2018 were identified, including: (1) surgery with curative intent, with or without supportive radiotherapy; (2) curative radiotherapy; (3) pharmacological treatments, including systemic treatment associated with surgery (neoadjuvant and/or adjuvant), radiotherapy (induction, concomitant or sequential) and/or treatments with palliative intent.

Surgery with curative intent included pneumonectomy, lobectomy and segmentectomy or wedge resection.

Radiotherapy complementary to surgery was differentiated into either preoperative radiotherapy (NRT) if administered prior to surgery with curative intent, or postoperative radiotherapy (ART) if administered after surgery with a curative intent. Curative radiotherapy considered SBRT and hypofractionated high-dose radiotherapy.

Systemic pharmacological treatments included both chemotherapy and biomarker-targeted therapies (ITK-ALK/ROS1, ITK-EFGR, immunotherapy and antiangiogenic therapy) and were classified according to their therapeutic group [34]. Pharmacological treatments were classified as follows:

- NACT ± RT: neoadjuvant treatment, with or without complementary radiotherapy, administered prior to surgery with a curative intent.
- ACT ± RT: adjuvant treatment, with or without complementary radiotherapy, initiated for a maximum of 10 weeks after surgery with curative intent [35].
- IND: induction therapy, started prior to the first session of radiotherapy with curative intent.
- CONCO: concomitant treatment, initiated within four weeks of the last session of radiotherapy with curative intent [36].
- SEQ: sequential therapy, started at least four weeks after the last session of radiotherapy with curative intent [36].
- Treatment for recurrent tumor: pharmacological treatment initiated more than 10 weeks after surgery with curative intent; after adjuvant treatment with a different therapeutic regimen; or from 12 weeks after completion of radiotherapy with curative intent [37] or adjuvant treatment [35] (with the same or different therapeutic regimen). Variations in pharmacological treatment that involved changes in active substances were classified as a subsequent line of treatment (second, third and so on).
- Treatments for unresectable tumor: pharmacological treatment in patients that did not receive surgery or radiotherapy with curative intent. Variations in pharmacological treatment that involved changes in active ingredients were classified as a subsequent line of treatment (second, third and so on).
- Patients who did not receive any of the therapeutic approaches above were considered as patients without systemic therapy.

The patients were classified into different groups and subgroups of treatment patterns according to the treatment types followed:

- Surgery: patients receiving surgery with curative intent, with or without complementary preoperative (NACT) and/or postoperative (ACT) pharmacological treatments, complementary preoperative, postoperative or radiotherapy (RT) alone. This category can also include the different lines of treatment for tumor recurrence.
- Radiotherapy: patients receiving radiotherapy with curative intent, with or without pharmacological treatments (IND, CONCO, SEQ). This category can also include different lines of treatment for recurrent tumors.
- Unresectable tumors: patients whose records show only pharmacological treatments with a palliative intent for inoperable disease.
- Without systemic therapy: patients with no record of any of the previous treatments.

For each treatment pattern, the patient’s vital status at the last available follow-up was classified as death or “censored” (the latter includes loss to follow-up with no additional records of procedures). We assessed survival according to year of treatment initiation (2014 vs. 2018). Survival time was defined as the period from diagnosis (defined as the first admission for causes related to the lung cancer or lung cancer surgery with curative intent or first administration of radiotherapy or antitumor drug) until notification of death (last follow-up in December 2021). Cause of death was not available in the datasets used due to confidentiality clauses.

Finally, we determined the mean cost of the first year of treatment per patient for those starting treatment in 2014 and in 2018 and the annual pharmaceutical spending in 2014 and 2018 by treatment pattern. To assess cost of procedure, we only included the unit price for curative surgery intervention and radiotherapy tariff according their treatment objective. Both correspond to unit price specified in the Official Gazette of the Government of Catalonia and reimbursed yearly by the Catalan Health Service according to the official fees approved. For procedures initiated in 2014 and 2018, we considered unit price from ORDER SLT/79/2014 and ORDRE SLT/150/2017, respectively. For oncological medicine costs, we considered the expenditure collected in the Datamart Billing Service, which uses the NHS reimbursed price.

### 2.4. Statistical Analysis

Descriptive statistical analysis was performed with categorical variables expressed as frequencies or proportions and continuous variables as means and standard deviations (SD). Survival analysis was performed using the Kaplan–Meier method, which includes the curve and the medians (95% confidence interval [CI]). The log-rank test was used to compare survival according to year of treatment initiation or therapeutic group. The chi-squared test was applied when categorical variables were compared, and the t-student test was applied in case of continuous quantitative variables comparison. Statistical analyses were carried out with the SPSS v18 software. To analyze the treatment pattern, a Sankey diagram was developed using the RStudio v4.0.1 tool.

### 2.5. Ethics

The Ethics Committee on Human and Animal Experimentation of the Universitat Autònoma de Barcelona approved the study (ref. CEEAH 4720).

## 3. Results

**Figure 1:**
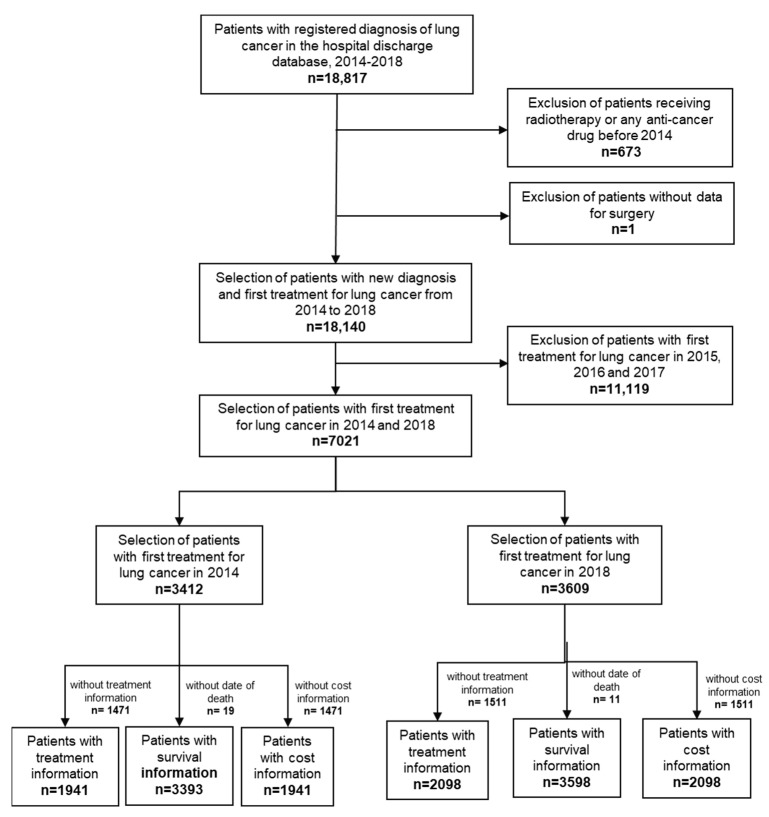
Flow diagram of lung cancer patients included in the study.

We identified 18,140 patients with a new diagnosis of lung malignancy between 2014 and 2018 (Figure 1). Table 1 shows only these patients with a first treatment for lung cancer in 2014 and 2018, and their characteristics according to their sex and age group. Among these patients, 3412 and 3609 were identified with a first treatment in 2014 and 2018, respectively. Most (79%, *n* = 2702 in 2014 and 77%, *n* = 2769 in 2018) were men, and over half (55%, *n* = 1858 in 2014 and 59%, *n* = 2139 in 2018) were aged 69 years or less.

Table 2 and Supplementary Table S1 present the distribution of the different treatment patterns group and subgroup initiated in 2014 and 2018 by age group. In 2014, 22% of patients underwent surgery with curative intent, compared to 24% in 2018. Curative radiotherapy was performed in 4% of patients in 2014, and this proportion remained steady in 2018. Patients who received pharmacological treatment directed at unresectable tumors represented 32% and 30% of patients in 2014 and 2018, respectively. Overall, there was a statistically significant change in the treatment pattern between 2014 and 2018. In particular, changes were observed in younger patients (<60 years). The percentage of these patients receiving surgery increased from 23% to 27% between 2014 and 2018, and the proportion of patients receiving pharmacological treatment for palliative intent decreased from 46% to 39%.

Table 3 and Supplementary Table S2 show the pharmacological regimes by treatment pattern for 2014 (*n* = 1358) and 2018 (*n* = 1347). Of the patients receiving adjuvant treatment, the percentage who received a pyrimidine analog with platinum increased from 64% in 2014 to 71% in 2018. Among those treated for unresectable disease, a smaller proportion followed a chemotherapy-based regimen in 2018 (82%) than in 2014 (91%). In contrast, the study period saw an increase in the percentage of patients who received an ICI as first-line treatment, reaching 9% by 2018. In 2014, 7% of patients received an EGFR-TKI, which is slightly more than the 6% who received this treatment in 2018. The proportion treated with ALK/ROS1-TKI reached 1% in 2018. Supplementary Figures S1 and S2 show the sequences of treatments throughout follow-up in patients initiating treatment in 2014 and 2018.

**Figure 2:**
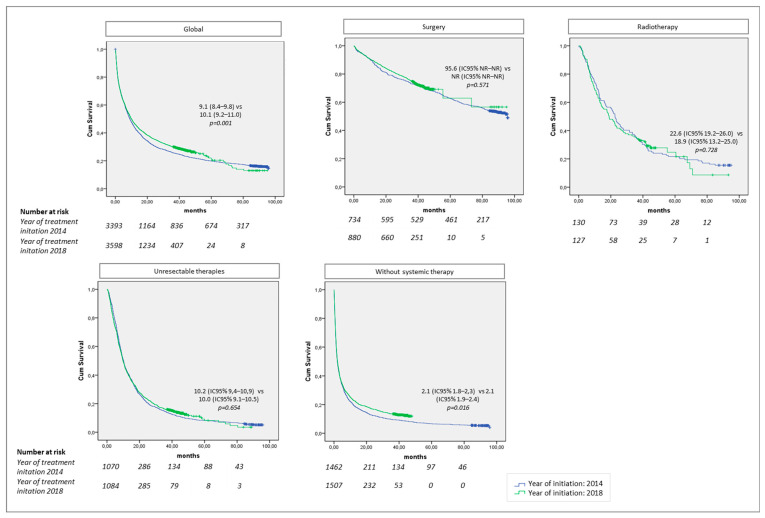
Kaplan–Meier curve for estimating overall survival for those who received first treatment in 2014 and 2018 by treatment pattern. NR: not reached. Global: patients who receive any of the treatments described below. Surgery: patients receiving surgery with a curative intent with or without neoadjuvant and/or adjuvant pharmacological treatment, and/or complementary radiotherapy; also includes different lines of treatment for tumor recurrence. Radiotherapy: patients with radiotherapy with a curative intent, with or without pharmacological treatments; also includes different lines of treatment for tumor recurrence. Unresectable tumor: patients who only receive pharmacological treatments with a palliative intent. Without systemic therapy: patients who do not receive any of the treatments described above.

Figure 2 compares the overall survival in patients who started treatment in 2014 versus 2018 by treatment pattern, and Supplementary Figure S3 by treatment pattern and age group. The median survival for patients with treatment imitation in 2014 was 9.1 months (95%CI 8.4–9.8) and 10.1 months (95%CI 9.2–11.0) for patients with treatment imitation in 2018; the differences between both groups were statistically significant. In the patterns that included surgery, the median survival was 95.6 months for those treated in 2014, whereas the median was not reached in patients who started treatment in 2018; no statistically significant differences were observed. The median survival in patients receiving radiotherapy was 22.6 months (95%CI 19.2–26.0) in 2014 and 18.9 months (95%CI 13.2–25.0) in 2018, and in those treated for unresectable disease, it was 10.2 months (95%CI 9.4–10.9) in 2014 and 10.0 months (95%CI 9.1–10.5) in 2018; these differences were not significant. The median survival in patients who did not receive any intervention was 2.1 months (95%CI 1.8–2.3) in 2014 and 2.1 months (95%CI 1.9–2.4) in 2018, and differences were significant. Regardless, median survivals by age group presented no difference between 2014 and 2018. The lack of treatment information in this group of patients could cause a bias linked to the treatment pattern description and/or their follow-up time. For patients treated for unresectable disease, survival rates by therapeutic groups and treatment initiation in 2014 and 2018 are shown in Supplementary Figure S4.

Table 4 presents the mean cost per patient of the first year of treatment beginning in 2014 and 2018. Mean costs for 2014 and 2018 were EUR 14,123 (SD 4327) and EUR 14,550 (SD 3880), respectively, for patients receiving surgery; EUR 4655 (SD 3540) and EUR 5873 (SD 6455) for patients receiving curative radiotherapy; and EUR 4723 (SD 7003) and EUR 6458 (SD 10,116) for patients receiving palliative treatments for unresectable tumors. Statistically significant differences were observed along time for each treatment patterns.

**Figure 3:**
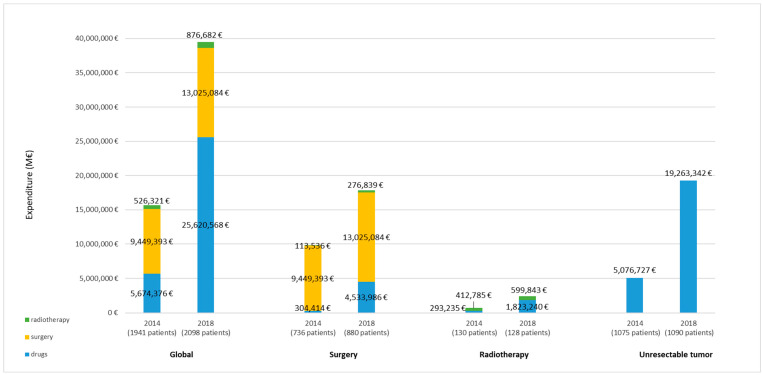
Pharmaceutical expenditure in 2014 and 2018 according to treatment pattern. Notes: The aggregated costs of the procedures were determined according to the treatment pattern followed in each patient. The costs for the year when each procedure was performed were imputed. Global: patients who receive any of the treatments described below. Surgery: patients receiving surgery with a curative intent with or without neoadjuvant and/or adjuvant pharmacological treatment, and/or complementary radiotherapy; also includes different lines of treatment for tumor recurrence. Radiotherapy: patients with radiotherapy with a curative intent, with or without pharmacological treatments; also includes different lines of treatment for tumor recurrence. Unresectable tumor: patients who only receive pharmacological treatments with a palliative intent.

Finally, the overall pharmaceutical spending in lung cancer showed an increase from 2014 to 2018, with aggregate values of EUR 15.7 million in 2014 and EUR 39.5 million in 2018 (Figure 3).

## 4. Discussion

The present study using real-world data and clinical practice outcomes of lung cancer treatment in relation to changes in treatment patterns, patient survival and costs provides a broader scope than previous studies, that have only studied the impact of certain interventions.

Firstly, the results showed, in absolute terms, an increase in the number of patients treated and a change in the treatment pattern between 2014 and 2018. In particular, we observed an increase in the proportion of patients undergoing surgery with a curative intent from 2014 to 2018 (from 22% to 24%), especially in patients under 60 (from 23% to 27%). In parallel, there was a slight reduction in the proportion treated for unresectable disease (32% to 30% globally and 46% to 39% in patients under 60 years). The main therapeutic goal of lung cancer treatment is complete tumor resection (R0) [10,38]. In medically inoperable patients with tumors over 5 cm in size, in a moderately central location or with comorbidities that may make surgery impossible, SBRT therapy or curative radiotherapy is recommended [10,38,39]. Therefore, the changes in strategy observed in this study could be related to a greater number of patients with resectable tumors and candidates for surgery, perhaps resulting from an earlier diagnosis thanks to the use of more sensitive imaging techniques [3,38], or due to an increase in patients diagnosed with lung adenocarcinoma [40,41,42], a tumor that is more likely to be excisable because it appears in the form of nodules or peripheral pulmonary tumors [12,37].

In patients with unresectable, previously untreated disease, 91% of those starting treatment in 2014 received a chemotherapy-based regimen. The most common ones (>10% of patients) consisted of platinum with antifolate, pyrimidine analogs (alone or in combination with platinum) and platinum, antifolate or taxane monotherapy. By 2018, the proportion of patients treated with chemotherapy decreased to 82%, a change that can be explained by the market entry of the earliest immuno-oncological agents as first-line treatments in 2017. In line with recommendations laid out in European and regional clinical practice guidelines for treating different tumor subtypes at advanced stages [10,11,35,43], the regimens started in 2018 and used in more than 10% of patients were combinations of platinum with pyrimidine analogs, podophyllotoxin derivatives or antifolate. In a retrospective observational study in the USA, patients with non-small cell lung carcinoma preferentially received the platinum-antifolate regimen. The combination of platinum plus taxane was also used as a standard treatment [28], although this is less common in our setting (9% of patients).

In our context, first-line ICIs were not reimbursed until 2017. By 2018, 9% of patients with unresectable tumors received an ICI as first-line treatment. This treatment was reimbursed only for the indication of non-small cell lung carcinoma with a PD-L1 expression of 50% or more [11], which represents 24% to 60% of patients with this histology [15,44]. However, in an observational study conducted in Canada, ICIs were rapidly adopted for different lines of treatment, especially after the drugs were approved in 2017 [44]. There, the approval of immunotherapy led to a more dramatic rise in the proportion of patients receiving these treatments for the first line, reaching around 17% in patients with advanced non-small cell lung carcinoma from 2016 to 2019 [45]. Thus, the initial uptake of immunotherapy in our context seems to have been slower than in Canada, although our analysis was not designed to identify or evaluate the speed of the adoption of immunotherapy.

In elderly patients (≥60 years), no significant changes in treatment patterns were observed. Age alone is not a contraindication for the prescription of pharmacological treatment. However, elderly patients more frequently present comorbidities (kidney, liver and bone marrow failure) and a lower functional reserve, which can condition the eligibility and tolerability of some treatments, especially combinations of immunotherapy and chemotherapy [11,39,46,47,48,49].

In relation to survival, treatment patterns that included surgery had clear benefits over curative radiotherapy or treatments aimed at unresectable disease, which was consistent with other observational studies [22,50]. The lack of statistically significant differences in survival for patients undergoing surgery between 2014 and 2018 suggests that there have been no changes in health outcomes or quality of care since cancer surgery in Catalonia was centralized in reference hospitals in 2012 [51,52]. Likewise, the survival results for those patients with curative radiotherapy do suggest that there have been significant changes in outcomes, even with the integration of SBRT in 2018.

In the context of unmet medical needs, the availability of new pharmacological treatments changes clinical practice. The inclusion of immunotherapy in different lines of treatment has special relevance, as clinical trials showed its effectiveness for long-term disease control and an improved overall survival compared to standard treatment [11,16,53,54]. In our context, the use of immunotherapy should entail survival benefits, especially in patients with recurrent or unresectable tumors at the time of diagnosis, but we did not observe any significant changes. However, the subgroup analysis among patients with unresectable disease showed better survival outcomes in patients treated with immunotherapy compared with chemotherapy. The proportion of patients treated with immunotherapy was small; it was likely that the uptake started in more severe cases, and the survival by treatment pattern was estimated within a larger set of interventions, which could mask the effect of these drugs. Therefore, it would be of interest to perform, in the future, an analysis with a longer time frame to monitor survival in a cohort with a greater representation of patients treated with immunotherapy.

In this context, we should also take into account that the patient survival could have been affected by the impact of the COVID-19 pandemic; in particular, those patients with a first treatment for lung cancer in 2018. Some studies reported a decrease in the overall cancer survival due to diagnostic, surgery or treatment initiation delays caused by the pandemic measures [55,56], and an increase in the risk of death due to infections in patients who were already vulnerable as a result of their immunocompromised status, their advanced age or comorbidities [57].

Although we did not observe any significant changes in the survival by the treatment pattern of patients who started treatment in 2014 compared to 2018, the mean cost per patient of the first year of treatment showed a significative rise. In Catalonia, the costs of surgery and radiotherapy correspond to unit prices defined by the procedures that make up highly complex hospital and specialized care, and, during the study period, these increased by 4% for surgery and 11% for radiotherapy. However, among patients with unresectable disease, we could deduce that the main reasons behind the increasing mean cost per patient are associated with the inclusion of immunotherapy and new targeted therapies.

Likewise, the health expenditure increased over the study period, as evidenced by the growing costs seen across the different treatment patterns, including drugs. All in all, the rise in cost seem rooted in the costs of drugs indicated for advanced tumor staging (in recurrence or unresectable at diagnosis), which comprise immunotherapy and target therapy in any line, with both groups linked to high prices. The overall budgetary impact of this rise was 153%: from EUR 15.7 million in 2014 to EUR 39.5 million in 2018. Although numerous measures have been implemented to manage prices and access to these treatments, costs have continued to climb. In the coming years, the economic impact is expected to mount even further with the approval of new pharmacological targeted treatments for patients who would previously have been treated with chemotherapy; the inclusion of regimens with multiple innovative, but expensive drugs; a longer duration of treatment, probably associated with a greater clinical benefit; and the positioning of generics and/or biosimilar drugs in subsequent lines of treatment, or even their replacement by new molecules.

Different studies have assessed the costs of managing lung cancer in specific European contexts [2,8,22,50,58,59,60,61]. However, it is difficult to compare their results with ours due to differences in the study approach, the unit prices of the procedures and the reimbursed prices of the drugs. In this sense, and as recommended by Andrade et al. [8], it could be useful to reach a consensus on the methodology used to estimate these costs.

This study has different limitations. First of all, we did not have access to some clinical information of interest, such as certain variables related to the tumor (histology, staging, biomarkers), the patient (functional status, tobacco consumption) or the treatment (indication of chemotherapy or radiotherapy), as these data were not available in the centralized registries. Patients were therefore categorized according to the procedures received. Another limitation is related to the selection of the study population, who were all patients admitted to hospital. In addition, ICD-coded clinical terms were modified during the study period due to the change from ICD-9 CM to ICD-10 CM.

Finally, some patients in our cohort could be included in a clinical trial in any line for lung cancer treatment, or could undergo surgery in a private hospital, but we lacked data on these circumstances in our healthcare registries. Should these information be available, we could more precisely identify their treatment pattern distribution and their survival and associated health-related costs.

## 5. Conclusions

This study is among the first to comprehensively analyze different lung cancer treatments based on clinical practice data in Catalonia using real-world data. Over the study period, there were changes in the treatment patterns applied, mostly apparent in younger patients. Likewise, we observed an increase in the overall costs of treating patients and especially in the pharmaceutical expenditure. These changes are mainly due to the reimbursement of new treatments targeted at biomarkers, such as immunotherapy and targeted therapy. Despite these changes, we did not observe differences in the overall patient survival. However, this outcome is expected to improve with time as a higher proportion of patients receive these innovative therapies, along with new molecules currently in clinical development. An analysis of a more recent time period will allow us to assess outcomes in a cohort where a higher proportion of patients are treated with innovative drugs.

## Figures and Tables

**Table 1:** Characteristics of patients diagnosed with malignant neoplasm of the lung, 2014 versus 2018.

	Year of Treatment Initiation 2014 (*n* = 3412)		Year of Treatment Initiation 2018 (*n* = 3609)		
	*n*	%	*n*	%	*p* Value *
Sex					0.013
Male	2702	79%	2769	77%	
Female	710	21%	840	23%	
Age groups					0.000
<60 years	805	24%	937	26%	
60–69 years	1053	31%	1202	33%	
70–79 years	1035	30%	1039	29%	
≥80 years	519	15%	431	12%	

* *p* value determined by chi square test.

**Table 2:** Treatment pattern group initiated in 2014 and 2018 by age group.

		2014 (*n* = 3412)		2018 (*n* = 3609)		
Age (Years)	Treatment Patterns	N	%	*n*	%	*p* Value *
Global	Surgery †	736	21.6%	880	24.4%	0.047
	Radiotherapy ‡	130	3.8%	128	3.5%	
	Unresectable tumor ^¶^	1075	31.5%	1090	30.2%	
	Without systemic therapy ^§^	1471	43.1%	1511	41.90%	
<60	Surgery †	181	22.5%	248	26.5%	0.039
	Radiotherapy ‡	27	3.4%	35	3.7%	
	Unresectable tumor ^¶^	371	46.1%	369	39.4%	
	Without systemic therapy ^§^	226	28.1%	285	30.4%	
60–69	Surgery †	281	26.7%	350	29.1%	0.203
	Radiotherapy ‡	44	4.2%	39	3.2%	
	Unresectable tumor ^¶^	382	36.3%	398	33.1%	
	Without systemic therapy ^§^	346	32.9%	415	34.5%	
70–79	Surgery †	241	23.3%	251	24.2%	0.841
	Radiotherapy ‡	44	4.3%	49	4.7%	
	Unresectable tumor ^¶^	269	26.0%	275	26.5%	
	Without systemic therapy ^§^	481	46.5%	464	44.7%	
≥80	Surgery †	33	6.4%	31	7.2%	0.286
	Radiotherapy ‡	15	2.9%	5	1.2%	
	Unresectable tumor ^¶^	53	10.2%	48	11.1%	
	Without systemic therapy ^§^	418	80.5%	347	80.5%	

* *p* value determined by chi square test. † Patients receiving surgery with a curative intent, with or without neoadjuvant and/or adjuvant pharmacological treatment, and/or complementary radiotherapy; also includes different lines of treatment for tumor recurrence. ‡ Patients with radiotherapy with a curative intent, with or without pharmacological treatments; also includes different lines of treatment for tumor recurrence. ^¶^ Patients who only receive pharmacological treatments with a palliative intent. ^§^ Patients who do not receive any of the treatments described above.

**Table 3:** Pharmacological treatments initiated in 2014 versus 2018 by treatment pattern.

	2014 (n = 1358)		2018 (n = 1347)	
	*n*	%	*n*	%
**Surgery**	**(*n* = 207)**		**(*n* = 194)**	
Pre-surgery and neoadjuvant therapies				
Platinum + pyrimidine analogues	22	43%	18	39%
Platinum + taxane	4	8%	9	19%
Platinum	13	25%	5	11%
Pyrimidine analogues	6	12%	4	9%
Other regimens *	6	12%	10	22%
Post-surgery and adjuvant therapies				
Platinum + pyrimidine analogs	109	64%	130	71%
Pyrimidine analogues	37	22%	8	4%
Platinum + antifolate	4	2%	17	9%
Platinum	10	6%	6	3%
Other regimens *	11	6%	24	13%
**Radiotherapy**	**(*n* = 76)**		**(*n* = 63)**	
Pre-radiotherapy and induction therapies				
Platinum	33	44%	5	8%
Vinca alkaloids	14	18%	8	13%
Platinum + vinca alkaloids	7	9%	17	27%
Taxane	8	11%	4	6%
Other regimens *	14	18%	29	46%
Post-radiotherapy and sequential therapies				
Taxane	1	100%	1	100%
**First-line treatment for unresectable tumors**	**(*n* = 1075)**		**(*n* = 1090)**	
Total chemotherapy	974	91%	893	82%
Antifolate + platinum	181	17%	211	19%
Platinum	153	14%	64	6%
Antifolate	140	13%	42	4%
Pyrimidine analogs	142	13%	54	5%
Platinum + pyrimidine analogs	132	12%	149	14%
Taxane	123	11%	30	3%
Platinum + podophyllotoxin derivative	1	0%	186	17%
Other chemotherapy regimens *	102	9%	157	14%
Immune checkpoint inhibitor	0	0%	98	9%
EGFR-TKI	76	7%	64	6%
ALK/ROS1-TKI	5	0%	13	1%
Other non-chemotherapy regimens *	20	2%	22	2%

Notes: table includes pharmacological regimens, including those targeting biomarkers (ICI, ITK-EGFR and ITK-ALK/ROS1) representing > 10% of the treatment strategy or >10 patients, with treatment initiation in 2014 or 2018. * See Supplementary Material Table S2 for other chemotherapy and non-chemotherapy regimens and their patient proportion.

**Table 4:** Mean cost (EUR) of the first year of treatment in 2014 and 2018.

		2014			2018		
		(*n* = 1941)			(*n* = 2098)		
Treatment Pattern	*n*	Mean	SD	*n*	Mean	SD	*p* Value ±
Surgery †	736	14,123	4327	880	14,550	3880	0.003
Radiotherapy ‡	130	4655	3540	128	5873	6455	0.051
Unresectable tumor ^¶^	1075	4723	7003	1090	6458	10116	0.000

SD: standard deviation. ± *p* value determined using Student’s *t*-test. † Patients receiving surgery with a curative intent, with or without neoadjuvant and/or adjuvant pharmacological treatment, and/or complementary radiotherapy; also includes different lines of treatment for tumor recurrence. ‡ Patients with radiotherapy with a curative intent, with or without pharmacological treatments; also includes different lines of treatment for tumor recurrence. ^¶^ Patients who only receive pharmacological treatments with a palliative intent.

## Data Availability

The data presented in this study are available on reasonable request from the corresponding author.

## Supplementary Materials

The following supporting information can be downloaded at: www.mdpi.com/article/10.3390/cancers14235791/s1, Figure S1: Sequences of procedures initiated in 2014; Figure S2. Sequences of procedures initiated in 2018; Figure S3. Kaplan-Meier curves for estimating overall survival who received first treatment in 2014 and 2018, by age group and treatment pattern. A: <60 years; B: 60-69 years; C: 70-79 years; D: >80 years; Figure S4. Kaplan-Meier curves for estimating overall survival among patients treated for unresecable tumors by therapeutic group and year of treatment initiation; Table S1: Treatment pattern subgroup initiated in 2014 and 2018, by age group; Table S2. Pharmacological treatments with <10% of utilization or >10 patients.
