# Supplementary material for: Changes in Treatment Patterns and Costs for Lung Cancer Have Not Resulted in Relevant Improvements in Survival: A Population-Based Observational Study in Catalonia
Source: Cancers (Basel). 2022 Nov 24;14(23):5791. doi: 10.3390/cancers14235791 (PMC9735431; doi:10.3390/cancers14235791)
Supplement: Supplementary file 1 [file cancers-14-05791-s001.zip › cancers-2008333-supplementary.pdf]

cancers MDPI

Supplementary material

Table S1. Treatment pattern subgroup initiated in 2014 and 2018, by age group.

Age (years)	Treatment patterns	2014 (n=3,412) n	2014 (n=3,412) %	2018 (n=3,609) n	2018 (n=3,609) %
Global	Surgery	494	14.5	643	17.%
	Surgery+RT	35	1.0	12	0.3
	NACT±RT +surgery	37	1.1	39	1.1
	NACT+ACT±RT+surgery	14	0.4	7	0.2
	ACT±RT+surgery	156	4.6	179	5.0
	IND+RT±SEQ	76	2.2	63	1.7
	CONCO+RT	0	0.0	0	0.0
	RT	54	1.6	65	1.8
	Unresectable tumor	1075	31.5	1090	30.2
	Without systemic therapy	1471	43.1	1511	41.9
<60	Surgery	99	12.3	167	17.8
	Surgery+RT	8	1.0	3	0.3
	NACT±RT +surgery	15	1.9	15	1.6
	NACT+ACT±RT+surgery	10	1.2	4	0.4
	ACT±RT+surgery	49	6.1	59	6.3
	IND+RT±SEQ	22	2.7	24	2.6
	CONCO+RT	0	0.0	0	0.0
	RT	5	0.6	11	1.2
	Unresectable tumor	371	46.1	369	39.4
	Without systemic therapy	226	28.1	285	30.4
60-69	Surgery	188	17.9	240	20.0
	Surgery+RT	14	1.3	3	0.2
	NACT±RT+surgery	13	1.2	19	1.6
	NACT+ACT±RT+surgery	2	0.2	3	0.2
	ACT±RT+surgery	64	6.1	85	7.1
	IND+RT±SEQ	33	3.1	19	1.6
	CONCO+RT	0	0.0	0	0.0
	RT	11	1.0	20	1.7
	Unresectable tumor	382	36.3	398	33.1
	Without systemic therapy	346	32.9	415	34.5
70-79	Surgery	177	17.1	210	20.2
	Surgery+RT	11	1.1	3	0.3
	NACT±RT+surgery	8	0.8	5	0.5
	NACT+ACT±RT+surgery	2	0.2	0	0.0
	ACT±RT+surgery	43	4.2	33	3.2
	IND+RT±SEQ	19	1.8	20	1.9
	CONCO+RT	0	0.0	0	0.0
	RT	25	2.4	29	2.8
	Unresectable tumor	269	26.0	275	26.5
	Without systemic therapy	481	46.5	464	44.7
≥80	Surgery	30	5.8	26	6.0
	Surgery+RT	2	0.4	3	0.7
	NACT±RT+surgery	1	0.2	0	0.0
	NACT+ACT±RT+surgery	0	0.0	0	0.0

ACT±RT+surgery	0	0.0	2	0.5
IND+RT±SEQ	2	0.4	0	0.0
CONCU+RT	0	0.0	0	0.0
RT	13	2.5	5	1.2
Unresectable tumor	53	10.2	48	11.1
Without systemic therapy	418	80.5	347	80.5

Note: NACT±RT: neoadjuvant treatment, with or without complementary radiotherapy, administered prior to surgery; can also include different lines of treatment for tumor recurrence. NACT+ACT±RT: neoadjuvant and adjuvant treatment, with or without complementary radiotherapy, administered prior to surgery; can also include different lines of treatment for tumor recurrence. ACT±RT: adjuvant treatment, with or without complementary radiotherapy, administered prior to surgery; can also include different lines of treatment for tumor recurrence. IND+RT±SEQ: induction therapy, started prior to the first session of radiotherapy with curative intent, with or without sequential therapy; can also include different lines of treatment for tumor recurrence. CONCO+RT: pharmacological treatment concomitant to radiotherapy with a curative intent; can also include different lines of treatment for tumor recurrence. Unresectable tumor: pharmacological treatments for an unresectable tumor. Without systemic therapy: Patients who do not receive any of the treatments described above.

Table S2. Pharmacological treatments with <10% of utilization or > 10 patients.

	2014	2018
**Pre-surgery and neoadjuvant therapies**	platinum+antifolate (6%); taxane (4%) and platinum+antifolate (6%)	platinum+podophyllotoxin derivative (9%); platinum+antifolate (4%); taxane (4%); pyrimidine analogues+taxane (2%); AAT (1%)
**Post-surgery and adjuvant therapies**	Taxane (4%); platinum+taxane (2%); platinum+antifolate (2%); antifolate (<1%).	platinum+podophyllotoxin derivative (5%); platinum+taxane (4%); taxane (2%); TOP1 (<1%); BRAF-TKI+MEK-TKI (<1%); antifolate (<1%)
**Pre-radiotherapy and induction therapies**	platinum+vinca alkaloids (9%); antifolate+platinum (7%); platinum+taxane (4%); antifolate (3%); platinum+pyrimidine analogs (<1%); EGFR-inh+platinum (<1%): antifolate+platinum+vinca alkaloids (<1%); AAT+platinum+pyrimidine analogs (<1%).	platinum+podophyllotoxin derivative (9%); platinum+taxane (9%); platinum+antifolate (9%); platinum+pyrimidine analogs+taxane (3%); platinum+pyrimidine analogs (5%); pyrimidine analogs (3%); ICI (2%); vinca alkaloids+ICI (2%); EGFR-inh (2%); platinum+podophyllotoxin derivative+vinca alkaloids (2%).
**First-line treatment for unresectable tumors - Other chemotherapy regimens**	platinum+vinca alkaloids (3%); vinca alkaloids (3%); TOP1 (2%), others+platinum(<1%), platinum+podophyllotoxin derivative (<1%), platinum+pyrimidine analogs+taxane(<1%), platinum+pyrimidine (<1%), analogs+vinca alkaloids (<1%), platinum+pyrimidine pyrimidine analogs+taxane (<1%), platinum+TOP1 (<1%), pyrimidine analogs+TOP1 (<1%), pyrimidine analogs+vinca alkaloids (<1%), pyrimidine analogs+taxane(<1%), pyrimidine analogs+taxane(<1%), pyrimidine analogs+TOP1 (<1%), taxane+antifolate (<1%), antifolate+platinum+taxane(<1%), platinum+vinca alkaloids(<1%) and antifolate+platinum+pyrimidine analogs (<1%).	platinum+taxane (9%); platinum (6%); pyrimidine analogs (5%) antifolate (4%), taxane (3%); platinum+antifolate (2%), podophyllotoxin derivative (1%), antifolate+ICI+platinum (<1%), others+platinum+podophyllotoxin derivative (<1%), platinum+podophyllotoxin derivative+TOP1 (<1%), platinum+vinca alkaloids (<1%), pyrimidine analogs+TOP1 (<1%), pyrimidine analogs+taxane (<1%), pyrimidine analogs+taxane (<1%), antifolate+platinum+pyrimidine analogs (<1%), platinum+pyrimidine analogs+TOP1 (<1%), platinum+TOP1 (<1%), platinum+taxane+vinca alkaloids (<1%), pyrimidine analogs+TOP1, platinum+pyrimidine analogs+taxane (<1%), TOP1 (<1%), vinca alkaloids (<1%), platinum+vinca alkaloids (<1%).
**First-line treatment for unresectable tumors - Other non-chemotherapy regimens**	AAT+antifolate (<1%), AAT+antifolate+platinum (<1%), AAT+TOP1+platinum (<1%), EGFR-inh+platinum (<1%), EGFR-inh+pyrimidine analogs (<1%), EGFR-inh+pyrimidine analogs+platinum+taxane (<1%), EGFR-TKI+antifolate (<1%), EGFR-inh+TOP1(<1%), platinum+vinca alkaloids (<1%), EGFR-inh(<1%) and AAT+platinum+pyrimidine analogs(<1%).	AAT (<1%), AAT+antifolate (<1%), AAT+antifolate+platinum (<1%), AAT+platinum+taxane (<1%), AAT+taxane (<1%), , EGFR-inh (<1%), EGFR-inh+platinum+pyrimidine analogs (<1%), EGFR-inh+pyrimidine analogs (<1%), taxane+AAT (<1%), AAT+platinum (<1%), AAT+platinum+pyrimidine analogs (<1%), EGFR-inh+platinum (<1%), mTOR-TKI (<1%), BRAF-TKI+MEK-TKI (<1%).

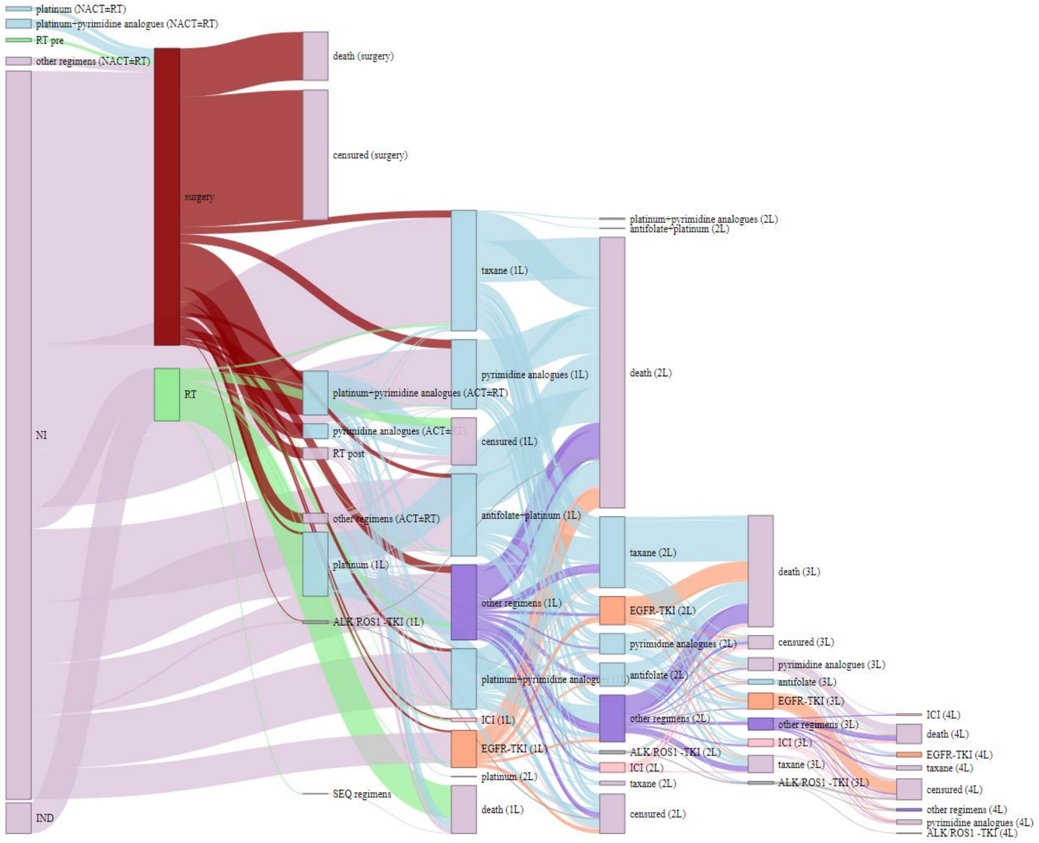

Figure S1. Sequences of procedures initiated in 2014. Note: figure includes pharmacological regimens, including those targeting biomarkers (ICI, ITK EGFR and ITK ALK/ROS1) representing > 10% of the treatment strategy or > 10 patients

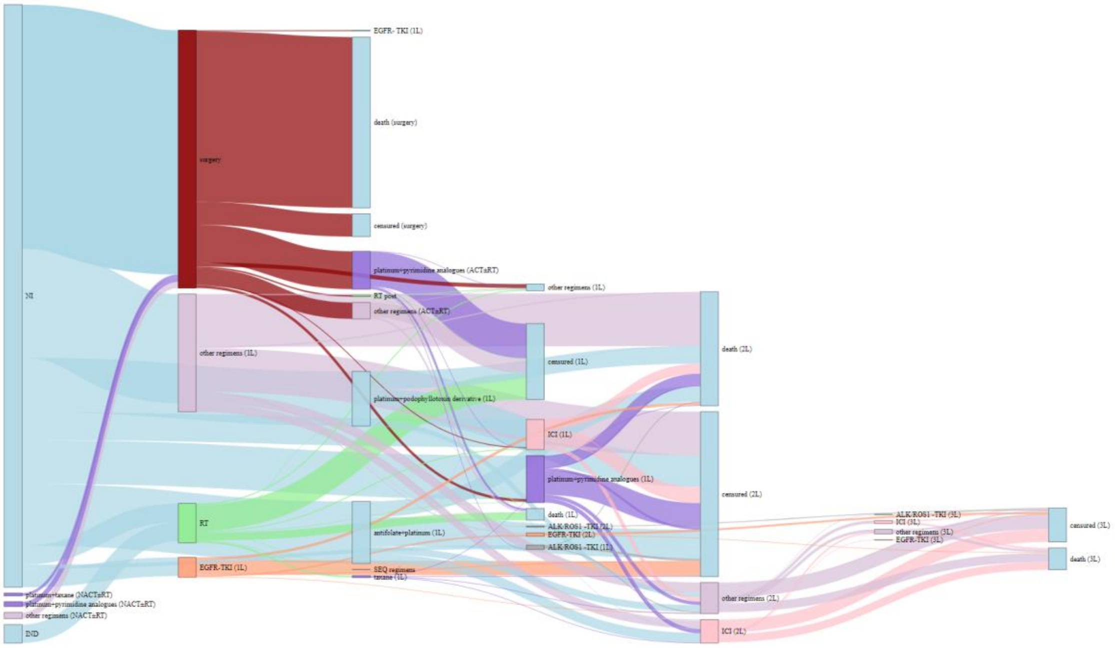

Figure S2. Sequences of procedures initiated in 2018. Note: figure includes pharmacological regimens, including those targeting biomarkers (ICI, ITK EGFR and ITK ALK/ROS1) representing > 10% of the treatment strategy or > 10 patients

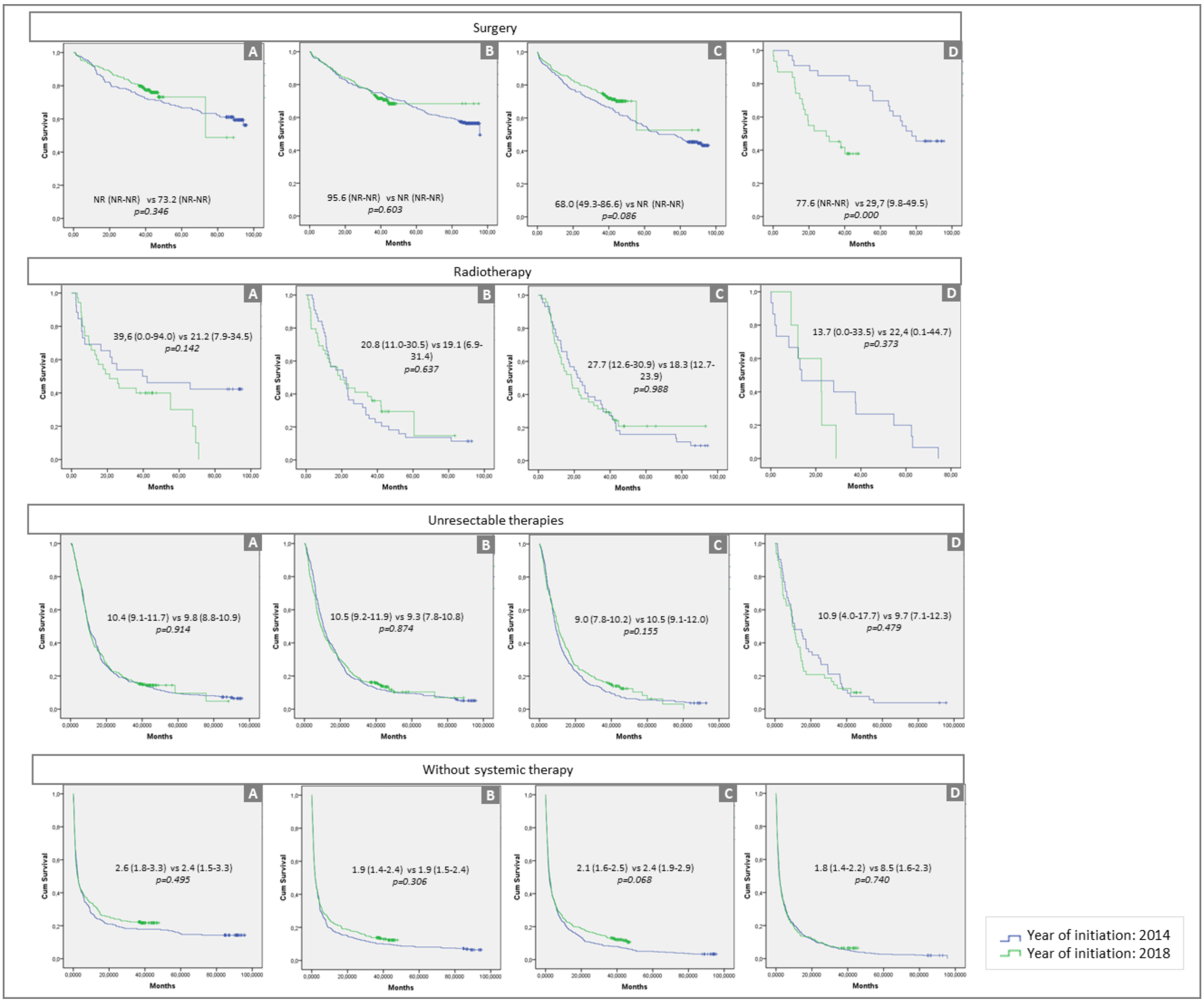

Figure S3. Kaplan-Meier curves for estimating overall survival who received first treatment in 2014 and 2018, by age group and treatment pattern. A: <60 years; B: 60-69 years; C: 70-79 years; D: >80 years. NR: not reached. Surgery: patients receiving surgery with a curative intent with or without neo-adjuvant and/or adjuvant pharmacological treatment, and/or complementary radiotherapy; also includes different lines of treatment for tumor recurrence.. Radiotherapy: patients with radiotherapy with a curative intent, with or without pharmacological treatments; also includes dif-ferent lines of treatment for tumor recurrence. Unresectable tumor: patients who only receive pharmacological treatments with a palliative intent. Without systemic therapy: patients who do not receive any of the treatments described above.

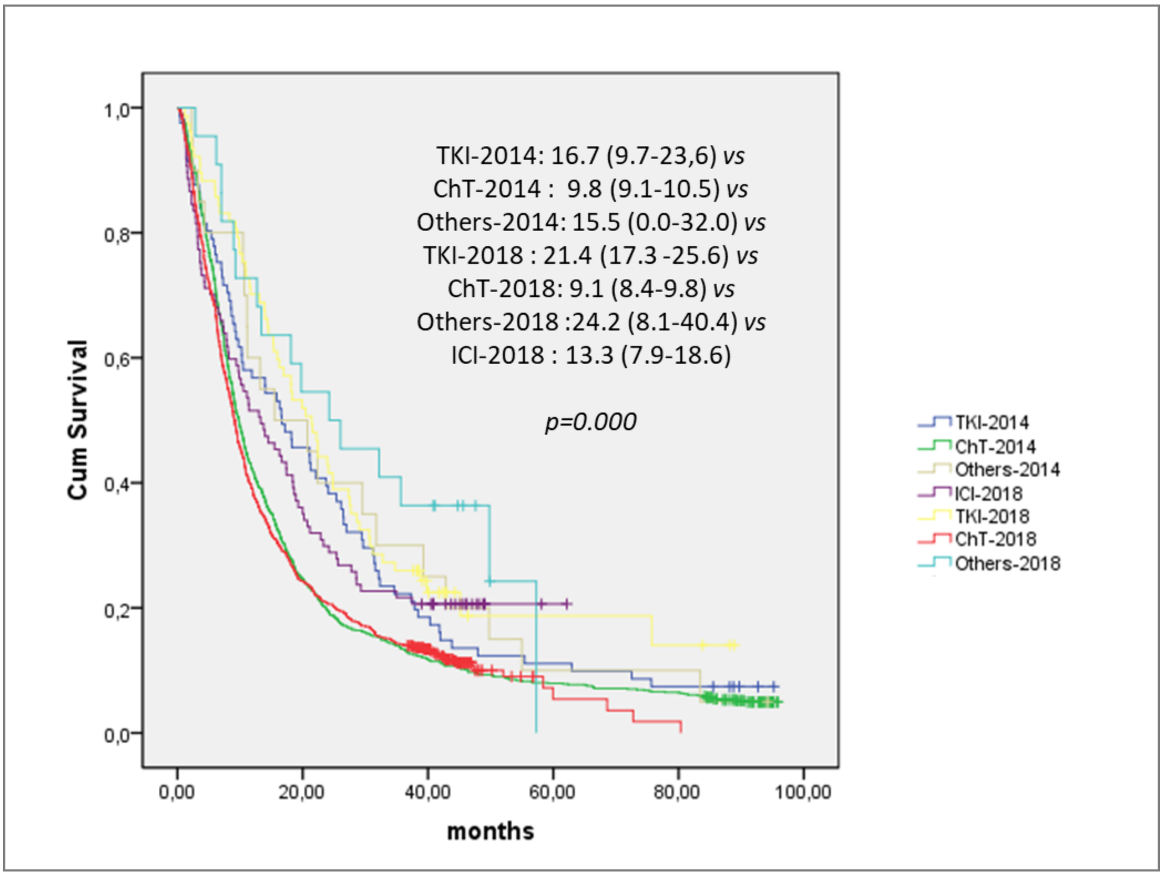

Figure S4. Kaplan-Meier curves for estimating overall survival among patients treated for unresecable tumors by therapeutic group and year of treatment initiation. Note: TKI: tirosin-kinassa inhibitors targeting EGFR mutations, ALK translocations and ROS-1 translocation; ChT: topoisomerase 1 inhibitors; antifolate, platinum, pyrimidine analogues, podophyllotoxin derivative, taxane, vinca alkaloids. Others: Anti-angiogenic therapy, TKI targeting MEK, BRAF and mTOR, EGFR inhibitors
